# Are clinicopathological features of the isthmic thyroid nodule different from nodules in thyroid lobes? A single center experience

**DOI:** 10.20945/2359-3997000000345

**Published:** 2021-04-12

**Authors:** Fatma Dilek Dellal, Oya Topaloglu, Husniye Baser, Ahmet Dirikoc, Afra Alkan, Aysegul Aksoy Altinboga, Ibrahim Kilinc, Reyhan Ersoy, Bekir Cakir

**Affiliations:** 1 Ankara City Hospital Department of Endocrinology and Metabolism Ankara Turkey Ankara City Hospital, Department of Endocrinology and Metabolism, Ankara, Turkey.; 2 Yildirim Beyazit University Medical Faculty Department of Endocrinology and Metabolism Ankara Turkey Yildirim Beyazit University Medical Faculty, Department of Endocrinology and Metabolism, Ankara, Turkey.; 3 Yildirim Beyazit University Medical Faculty Department of Biostatistics Ankara Turkey Yildirim Beyazit University Medical Faculty, Department of Biostatistics, Ankara, Turkey.; 4 Yildirim Beyazit University Medical Faculty Department of Pathology Ankara Turkey Yildirim Beyazit University Medical Faculty, Department of Pathology, Ankara, Turkey.; 5 Ankara City Hospital Department of General Surgery Ankara Turkey Ankara City Hospital, Department of General Surgery, Ankara, Turkey.

**Keywords:** Isthmus, ultrasonography, cytology, histopathology, thyroid cancer

## Abstract

**Objectives:**

Thyroid nodules located in isthmus were found less prevalent, although papillary thyroid cancer in this location was reported to be more aggressive behaviour in some studies. Our aim was to evaluate hormonal,ultrasonographic, and cytopathologic features of nodules located in isthmus (isthmic nodules).

**Subjects and methods:**

Patients who underwent thyroidectomy between 2006-2014 reviewed retrospectively. Hormonal, ultrasonographic, and cytopathologic features compared between patients with isthmic (Group-1) and with lober (non-isthmic, Group-2) nodules.

**Results:**

Group-1 and Group-2 consisted of 251 and 2076 patients, respectively. 260 isthmic (5.5%) and 4433 non-isthmic (94.5%) nodules were compared.However,most ultrasonographical features such as presence of microcalcification and halo, diameters, echogenicity, texture, margin, and vascularity were similar between groups, macrocalcification rate was lower in isthmic nodules (18.8%, 25.9%; p = 0.012). Cytologic results were also similar.Although malignancy rate was lower in isthmic nodules (6.2%, 12.5%; p = 0.002), type of thyroid cancer was similar in isthmic and non-isthmic nodules.When malignant isthmic (n = 16,2.8%) and malignant non-isthmic nodules (n = 553, 97.2%) were compared, diameter and type of tumor, lymphovascular and capsular invasions, extrathyroidal extension and multifocality rates were not statistically significant.Malignant isthmic nodules (n = 16, 6.2%) had smaller size [10.1 (7.5-34.5) mm, 19.95 (8.4-74.1) mm; p = 0.002], and higher hypoechogenicity rate (31.3%, 5.7%, p = 0.003) compared to benign isthmic nodules (n = 244, 93.8%). Negative predictive value was higher and positive predictive value was lower in isthmic nodules compared to non-isthmic nodules (p = 0.033, p = 0.047, respectively).

**Conclusion:**

Isthmic nodules appear to be indolent because of having lower malignancy rate. FNAB might be required in isthmic nodules even if it has relatively small size.The surgery with limited extent or follow-up might seem to be reliable in the management of patients having isthmic nodules especially with indeterminate cytology.

## INTRODUCTION

The prevalence of having a thyroid nodule is 3-7% by palpation in population (1), and the lifetime risk for developing a palpable thyroid nodule is estimated as 10% (2). Nodule diagnose has been increased in clinical practice, especially with advancement of diagnostic techniques. Its prevalence is reached to 19-68% by high-resolution ultrasound (US) (2,3).

Thyroid fine needle aspiration biopsy (FNAB) is the most accurate and reliable diagnostic technique for diagnosis of malignancy in thyroid nodules. It helps to select candidates for surgery, however this is an invasive procedure. US is a non-invasive, sensitive, cheap, and easy method for evaluating the nodules. Some US features were found to be associated with higher malignancy risk (4). These are hypoechogenity, solid texture, micro- and/or macrocalcification, lack of peripheral halo, irregular margin, taller than wider shape, and increased vascularity (5). Also, Thyroid Imaging Reporting and Data System (TIRADS) is the assessment tool that categorizes thyroid nodules and stratifies their malignancy risk usually using a score according to their US features (6). Recently, whether nodule localization is a malignancy predictor like these suspicious US features has become the focus of interest (7–10).

The isthmus is the smallest part of thyroid gland which connects right and left lobes. Prevalence of thyroid nodules located in isthmus was found as 4.2-6.4% (7,9,11). The incidence of isthmic papillary thyroid cancer (PTC) ranges from 1% to 12.3% in different studies (12–21). Although PTC had an indolent course, isthmic PTCs were reported to have more aggressive behaviour, including multifocality, capsular invasion, and frequently having metastasis to lymph nodes in some studies (12,16). The features of other histopathologic types of thyroid carcinomas arising from isthmus are unknown. There is no specific suggestion for diagnosis and follow-up of isthmic thyroid nodules in clinical guidelines. There have been a few studies regarding cytologic and ultrasonographic features of isthmic thyroid nodules in the literature. Therefore, in the present study, our aim is to evaluate whether thyroid nodules located in the isthmus are different from located in thyroid lobes according to their clinicopathological features.

## SUBJECTS AND METHODS

### Patient selection

The records of 2441 patients who underwent thyroidectomy between 2006-2014 were screened for this study. Exclusion criteria were previous history of thyroidectomy and radiotherapy to head and neck region, absence data of cytopathological and histopathological features, presence of nodules without coupled cytopathologic and histopathological results, and incidentally found malignant parenchymal lesions. Finally, 2327 patients were enrolled. Patients divided into two groups: having isthmic nodules (Group-1) and non-isthmic nodules (located in right or left thyroid lobes) (Group-2). Group-1 had 251 (10.8%) and Group-2 had 2076 (89.2%) patients. Demographic features, laboratory, cytology and histopathology results were compared between groups. Furthermore, the tumour characteristics of malignant nodules such as the type, size, multifocality, vascular invasion, lymphatic invasion, capsular invasion, extrathyroidal extension, lymph node metastasis and TNM stages (22), radioactive iodine (RAI) administration and RAI doses, and stimulated thyroglobuline (Tg) levels on 6th month of malignant patients were recorded.

Isthmic and non-isthmic nodules were compared in terms of US, cytopathologic and histopathologic features. Also, malignant nodules separated into isthmic and nonisthmic. Furthermore, isthmic nodules were analysed in two groups as benign and malignant nodules.

Local Ethical Committee of the School of Medicine, Yildirim Beyazit University approved this study.

### Laboratory

Serum thyroid stimulating hormone (TSH), free triiodothyronine (fT3), free thyroxine (fT4), anti-thyroid peroxidase (anti-TPO), anti-Tg antibody, and Tg measurements were made by chemiluminescence methods (Immulite 2000, Diagnostic Products Corporation, Los Angeles, CA, USA, and the UniCel DxI 800, Beckman Coulter, CA, USA). Reference ranges for TSH, fT3, fT4, Tg were 0.4 - 4.0 uIU/mL, 1.57-4.71 pg/mL, 0.61-1.12 ng/dL, and 0-78 ng/mL, respectively. Anti-TPO higher than 10 U/mL and anti-Tg higher than 30 U/mL were interpreted as positive. Patients on levothyroxine treatment and/or patients with a serum TSH above reference ranges were defined to have hypothyroidism. Patients taking antithyroid medication and/or patients with a serum TSH below reference ranges were considered to have hyperthyroidism. Patients with TSH levels in normal ranges were accepted as euthyroid.

### Imaging studies

Thyroid US was carried out with a high resolution ultrasound instrument (Esaote color Doppler US (Taipei, Taiwan)) equipped with a 5.5-12.5 MHz linear probe. Isthmic nodule was defined when a center of nodule was localized lateral border of isthmus by drawing two imaginary lines perpendicular to skin surface from most lateral borders of trachea on the transverse scan compatible with Hahn and cols. (17).

US feaures of nodules such as diameters, presence of calcification (micro-, macrocalcification), taller than wider shape, echogenicity (isoechoic, hypoechoic, hyperechoic), texture (solid, cystic/mixed), presence of halo, presence of cystic degeneration, margins (irregular, regular), vascularity (peripheral, central) were noted. Large thyroid nodule was defined as one of three dimensions of the nodule is 40 mm or more in US evaluation. Furthermore, TIRADS categories of nodules were recorded. For TIRADS assessment, suspicious US features for malignant nodule(solid structure, low or very low echogenicity, irregular or microlobular borders, microcalcifications, and taller-than-wider) was scored as 1 for presence or 0 for absence. According to sum of each scores of suspicious features, TIRADS groups determined as 5 groups: Category 3, 4a, 4b, 4c, and 5 if sum of the scores were 0, 1, 2, 3 or 4, and 5; respectively (23).

### Fine needle aspiration biopsy, cytopathology and histopathology

US-guided Fine Needle Aspiration Biopsy (FNAB) was performed by an experienced clinician by a 27 gauge needle and 20 mL volume syringe (Logic Pro 200 GE US machine and 7.5 MHz probe). FNAB was carried out on all nodules with a size of 1 cm or more. When there were clinical risk factors (family history of thyroid cancer) and/or suspicious US features, it was performed on nodules under 1 cm. Cytologic materials were categorized as nondiagnostic, benign, atypia of undetermined significance/follicular lesion of undetermined significance (AUS/FLUS), follicular neoplasm/suspicious for follicular neoplasm (FN/SFN), suspicious for malignancy and malignant according to Bethesda classification system (24).

### Statistical analysis

The distributions of continuous variables such as age were examined by Shapiro-Wilk’s test and normality plots. All continuous and discrete variables were reported as median (min-max), while categorical variables were expressed by frequency (%).

Patients with isthmic and non-isthmic nodules were compared by Mann-Whitney U test for continuous and discrete variables and by chi-square tests for categorical variables. In the case of more than 50% of cells have expected count less than 5, the p value of Monte-Carlo simulation based on 10000 samples were given for categorical variables.

The diagnostic accuracy measures of the cytology was examined by considering the Bethesda categories > II (AUS/FLUS, FN, SFN, suspicious for malignancy) as malignant in isthmic and non-isthmic nodules. Nondiagnostic cytologic results were excluded from the analysis. The diagnostic accuracy measures were provided with their 95% CIs determined by Wilson score method. Diagnostic accuracy measures were compared by Pearson chi-square, Yates chi-square or Fisher’s exact test, where appropriate.

A p value < 0.05 was considered as statistically significant. Wilson’s score CIs was obtained by using PropCIs package (25) in RStudio Software (Version 1.3.959) (26). All other analyses were performed via IBM SPSS Statistics 22.0 (IBM Corp. Released 2012. IBM SPSS Statistics for Windows, Version 22.0. Armonk, NY: IBM Corp.).

## RESULTS

The age and gender distributions were similar between Group-1 (n = 251) and Group-2 (n = 2076) (p > 0.05). Although TSH level was found as high significantly in Group-2 compared to Group-1 (p < 0.001), fT3 and fT4 levels were higher in Group-1 than Group-2 (p = 0.031 and p = 0.007, respectively). Anti-Tg positivity was found as more in Group-1 (p = 0.046). The presence of hyperthyroidism, the isthmus dimension, and the proportion of thyroidectomy decision because of having a large nodule were significantly higher in Group-1 compared to Group-2 (p < 0.05) (Table 1).

**Table 1:** Clinical and demographic features of patients in Group-1 and Group-2

	Group-1 (Isthmic ) [n = 251]	Group-2 (Non-isthmic) [n = 2076]	p
	Median (min-max) n (%)	Median (min-max) n (%)	
Age, years	51 (23-85)	50 (18-84)	0.054
Gender, F	198 (78.9)	1612 (77.6)	0.657
TSH^a^, µIU/mL	0.60 (0.001-11.00)	**1.10 (0.001-51.10)**	**<0.001**
fT3^b^, pg/mL	**3.31 (1.40-9.16)**	3.28 (1.03-66.0)	**0.031**
fT4^c^, ng/dL	**1.20 (0.48-4.20)**	1.15 (0.07-10.70)	**0.007**
Thyroid hormonal status			
Euthyroid	184 (73.3)	1614 (77.7)	0.113
Hypothyroid	3 (1.2)	**107 (5.2)**	**0.008**
Hyperthyroid	**64 (25.5)**	355 (17.1)	**0.001**
Anti-TPO positivity	45 (19.1)	396 (21.9)	0.332
Anti-Tg positivity	**60 (26.8)**	375 (21.0)	**0.046**
Isthmus dimension^d^, mm	**14.5 (2-40)**	4.6 (0-40)	**<0.001**
Thyroidectomy indication			
Large nodule	**118 (47.0)**	640 (30.8)	**<0.001**
Hyperthyroidism	29 (11.6)	235 (11.3)	0.912
Cytology	81 (32.3)	**992 (47.9)**	**<0.001**
Parathyroid pathology	3 (1.1)	38 (1.8)	0.616
Other/unknown	20 (8.0)	171 (8.2)	0.980
Cytology subgroups causing thyroidectomy decision			0.375
Nondiagnostic	16 (19.8)	226 (22.8)	
AUS/FLUS and suspicious US findings	34 (42.0)	369 (37.2)	
FN/SFN	10 (12.3)	92 (9.3)	
Suspicious for malignancy	15 (18.5)	163 (16.4)	
Malignant	6 (7.4)	142 (14.3)	
Lymphocytic thyroiditis in histopathology	54 (21.5)	**573 (27.6)**	**0.040**

TSH: thyrotropin; fT3: free triiodothyronine; fT4: free thyroxine; Anti-TPO: anti-thyroid peroxidase antibody; Anti-Tg: anti-thyroglobulin antibody; AUS/FLUS: atypia of undetermined significance/follicular lesion of undetermined significance; US: ultrasound; FN/SFN: follicular neoplasm/suspicious for follicular neoplasm.

a n = 250 and n = 2069 for isthmic and non-isthmic, respectively.

b n = 2062 for non-isthmic patients.

c n = 2072 for non-isthmic patients.

d n = 247 and n = 2042 for isthmic and non-isthmic, respectively.

Totally 260 isthmic nodules from 251 patients in Group-1 and 4433 non-isthmic nodules from 2076 patients in Group-2 were compared according to US findings, cytology and histopathology results (Table 2). US findings, exception of presence of macrocalcification, were similar between groups (p > 0.05). Presence of macrocalcification was higher in non-isthmic nodules (p = 0.012). Furthermore, cytology findings were similar between groups (p > 0.05). Histopathology was determined as malignant in 16 isthmic nodules (6.2%) and 553 non-isthmic nodules (12.5%) (p = 0.002). The ratio of nodule detected as PTC histopathologically was higher in non-isthmic nodules compared to isthmic nodules (11.7% vs 5.8 %) (p = 0.003).

**Table 2:** Ultrasonographic features, fine needle aspiration biopsy and histopathology results of nodules in Group-1 and Group-2

		Group-1 (Isthmic) [n = 260]	Group-2 (Non-isthmic) [n = 4433]	p
		Median (min-max) n (%)	Median (min-max) n (%)	
Antero-Posterior Diameter^a^, mm		12.2 (3.0-58.5)	12.4 (0.1-70.2)	0.179
Transverse Diameter^b^, mm		**17.0 (5.9-74.9)**	14.9 (2.9-256.0)	**0.046**
Longitudinal Diameter^c^, mm		19.55 (4.0-74.1)	17.6 (3.9-113.0)	0.618
TIRADS categories				0.383
3		2 (0.7)	57 (1.3)	
4a		55 (21.2)	962 (21.7)	
4b		124 (47.7)	1891 (42.7)	
4c		79 (30.4)	1501 (33.8)	
5		0 (0.0)	22 (0.5)	
Taller than wider shape		33 (12.9)	756 (17.4)	0.064
Absence of peripheral halo		189 (72.7)	3162 (71.3)	0.636
Microcalcification		85 (32.7)	1590 (35.9)	0.299
Macrocalcification		49 (18.8)	**1146 (25.9)**	**0.012**
Echogenicity				0.109
Isoechoic		150 (58.1)	2248 (52.0)	
Hypoechoic		19 (7.4)	442 (10.2)	
Iso-hypoechoic		89 (34.5)	1634 (37.8)	
Texture, Solid		251 (96.5)	4219 (95.2)	0.392
Irregular margins		166 (63.8)	2641 (59.6)	0.172
Vascularity, peripheral		20 (76.9)	441 (73.3)	0.851
Cytology				0.097
	Non-diagnostic	55 (21.2)	1173 (26.5)	
	Benign	152 (58.3)	2407 (54.2)	
	AUS/FLUS	29 (11.2)	459 (10.4)	
	FN/SFN	8 (3.1)	83 (1.9)	
	Suspicious for malignancy	13 (5.0)	172 (3.9)	
	Malignant	3 (1.2)	139 (3.1)	
Histopathology, Malignant		16 (6.2)	**553 (12.5)**	**0.002**
Type of thyroid cancer				
	Papillary cancer	15 (5.8)	**518 (11.7)**	**0.003**
	Follicular cancer	0 (0.0)	20 (0.5)	0.624
	Hurthle cell cancer	1 (0.4)	15 (0.3)	0.599
	Benign	**244 (93.8)**	3880 (87.5)	**0.002**
PTC variants, classical		9 (60.0)	333 (64.3)	0.946

TIRADS: Thyroid Imaging Reporting and Data System; AUS/FLUS: atypia of undetermined significance/follicular lesion of undetermined significance; FN/SFN: follicular neoplasm/suspicious for follicular neoplasm; PTC: papillary thyroid cancer

a n = 250 and n = 2069 for isthmic and non-isthmic, respectively.

b n = 2062 for non-isthmic patients.

c n = 256 and n = 4392 for isthmic and non-isthmic, respectively.

The demographic and clinical characteristics of the patients with malignant isthmic and non-isthmic nodules were given in Table 3. Groups had 16 and 503 malignant patients, respectively. Age, gender distributions and other laboratory findings except from anti-Tg positivity were similar (p > 0.05). It was significantly higher in isthmic group (p = 0.010). Furthermore, TNM classifications of malignant nodules and numbers of patients with RAI treatment were not different in two groups (p > 0.05). Stimulated Tg level was lower in patients with malignant isthmic nodules (p = 0.015).

**Table 3:** Clinicopathological features of patients with malignant isthmic and malignant non-isthmic nodules

Malignant nodules	Isthmic [n = 16]	Non-isthmic [n = 553]	p
	Median (min-max) n (%)	Median (min-max) n (%)	
Age, years	49.5 (31-68)	48 (19-84)	0.874
Gender, F	13 (81.3)	400 (79.5)	1.000
TSH^a^, µIU/mL	1.96 (0.16-11.00)	1.60 (0.002-24.00)	0.391
fT3^b^, pg/mL	3.12 (2.39-4.03)	3.20 (1.03-7.68)	0.949
fT4^c^, ng/dL	1.29 (0.96-2.20)	1.17 (0.26-4.10)	0.127
Thyroid hormonal status			0.268
Euthyroid	11 (68.8)	427 (84.9)	
Hypothyroid	2 (12.5)	36 (7.2)	
Hyperthyroid	3 (18.7)	40 (7.9)	
Anti-TPO positivity	5 (33.3)	110 (25.8)	0.551
Anti-Tg positivity	**8 (57.1)**	104 (24.3)	**0.010**
Number of malignant focus^d^	1 (1-4)	1 (1-15)	0.378
Multifocality	4 (25.0)	197 (39.7)	0.354
Lymph node metastasis	1 (6.3)	52 (10.4)	1.000
T classification			0.053^e^
T1a	6 (37.5)	134 (30.8)	
T1b	8 (50.0)	122 (28.0)	
T2	2 (12.50)	76 (17.4)	
T3	0 (0.0)	99 (22.8)	
T4a	0 (0.0)	2 (0.5)	
T4b	0 (0.0)	2 (0.5)	
TNM stage			0.126^f^
Stage I	15 (93.8)	366 (73.4)	
Stage II	1 (6.2)	47 (9.4)	
Stage III	0 (0.0)	78 (15.6)	
Stage IVa	0 (0.0)	7 (1.4)	
Stage IVb	0 (0.0)	1 (0.2)	
RAI treatment	14 (93.3)	452 (97.8)	0.299
RAI dose, >100 mCi	5 (35.7)	238 (53.0)	0.315
Stimulated Tg level on 6th month^e^, ng/mL	0.235 (0.02-6.06)	**2.170 (0.02-505.00)**	**0.015**

TSH: thyrotropin; fT3: free triiodothyronine; fT4: free thyroxine; Anti-TPO: anti-thyroid peroxidase antibody; Anti-Tg: anti-thyroglobulin antibody; TNM: tumour, node, metastasis, RAI: radioactive iodine.

a n = 15 and n = 502 for isthmic and non-isthmic, respectively.

b n = 499 for non-isthmic.

c n = 502 for non-isthmic.

d n = 496 for non-isthmic.

e n = 12 and n = 437 for isthmic and non-isthmic, respectively.

f Monte Carlo simulation results based on 10000 samples.

US features, cytology and histopathology results of malignant isthmic and non-isthmic nodules were given in Table 4. The anti-Tg positivity and cytology of suspicious for malignancy were significantly higher in malignant isthmic nodules (p < 0.05). All of the ultrasonographic findings and TIRADS categories were similar between groups. Furthermore, tumor diameter, presence of lymphatic, vascular and capsular invasions and extrathyroidal extension were not different.

**Table 4:** Ultrasonography features, fine needle aspiration biopsy and histopathology results of nodules in malignant isthmic and malignant non-isthmic groups

Malignant nodules		Isthmic [n = 16]	Non-isthmic [n = 552]	p
		Median (min-max) n (%)	Median (min-max) n (%)	
Anti-TPO positivity		5 (33.3)	122 (26.1)	0.555
Anti-Tg positivity		**8 (57.1)**	115 (24.6)	**0.011**
Antero-posterior diameter^a^, mm		7.45 (4.30-20.20)	10.50 (0.10-54.00)	0.071
Transverse diameter^b^, mm		9.20 (6.40-32.00)	12.05 (3.90-67.90)	0.493
Longitudinal diameter^c^, mm		10.05 (4.00-34.50)	13.50 (3.90-84.90)	0.122
TIRADS categories				0.943
3		0 (0.0)	2 (0.3)	
4a		3 (18.8)	96 (17.4)	
4b		5 (31.2)	176 (31.9)	
4c		8 (50.0)	267 (48.4)	
5		0 (0.0)	11 (2.0)	
Taller than wider shape		2 (13.3)	117 (22.4)	0.540
Absence of peripheral halo		13 (81.3)	405 (73.4)	0.579
Microcalcification		6 (37.5)	254 (46.0)	0.675
Macrocalcification		2 (12.5)	186 (33.7)	0.132
Echogenicity				0.161
Isoechoic		2 (12.4)	193 (35.6)	
Hypoechoic		3 (21.3)	125 (23.1)	
Iso-hypoechoic		9 (56.3)	224 (41.3)	
Texture, Solid		15 (93.8)	542 (98.2)	0.272
Irregular margins		10 (62.5)	352 (63.8)	1.000
Vascularity, peripheral		2 (100.0)	65 (61.9)	0.527
Cytology				
	Non-diagnostic	0 (0.0)	79 (14.3)	0.146
	Benign	0 (0.0)	87 (15.8)	0.149
	AUS/FLUS	4 (25.0)	99 (17.9)	0.509
	FN/SFN	2 (12.5)	27 (4.9)	0.196
	Suspicious for malignancy	**8 (50.0)**	126 (22.8)	**0.031**
	Malignant	2 (12.5)	134 (24.3)	0.381
Type of thyroid cancer				0.420
	Papillary cancer	15 (93.8)	518 (93.9)	
	Follicular cancer	0 (0.0)	20 (3.6)	
	Hurthle cell cancer	1 (6.2)	14 (2.5)	
PTC variants, classical		9 (60.0)	333 (64.3)	0.946
Tumor diameter^d^, mm		12.0 (5.0-35.0)	12.0 (1.0-90.0)	0.859
Microcarcinoma		6 (37.5)	219 (40.0)	1.000
Lymphatic extension		0 (0.0)	16 (2.9)	1.000
Vascular invasion		1 (6.3)	38 (6.9)	1.000
Capsular invasion		6 (37.5)	205 (37.5)	1.000
Extrathyroidal extension		0 (0.0)	96 (17.6)	0.087
Lymphocytic thyroiditis in histopathology		8 (50.0)	195 (35.3)	0.346

Anti-TPO: anti-thyroid peroxidase antibody; Anti-Tg: anti-thyroglobulin antibody; TIRADS: Thyroid Imaging Reporting and Data System; AUS/FLUS: atypia of undetermined significance/follicular lesion of undetermined significance; FN/SFN: follicular neoplasm/suspicious for follicular neoplasm; PTC: papillary thyroid cancer.

a n = 548 for non-isthmic.

b n = 15 and n = 524 for isthmic and non-isthmic, respectively.

c n = 551 for non-isthmic.

d n = 547 for non-isthmic.

Clinical and ultrasonographic features of malignant and benign isthmic nodules were presented in Table 5. Antero-posterior and transverse diameters were significantly smaller in malignant isthmic nodules (p < 0.05). TIRADS categories and US features exception of echogenicity were similar between two groups (p > 0.05). Hypoechoic feature was more prominent in malignant isthmic nodules compared to benign ones (p = 0.003). In cytology of malignant isthmic nodules, benign and ND results were not detected (p < 0.05). All isthmic nodules with non-diagnostic or benign cytology were diagnosed as benign histopathologically. AUS/FLUS and FN/SFN rates were similar between two groups (p = 0.089, p = 0.081; respectively). Lymphocytic thyroiditis in histopathology was significantly higher in the malignant isthmic nodules (p < 0.05).

**Table 5:** Clinical and ultrasonographic features of malignant and benign isthmic nodules

		Malisgnant [n = 16]	Benign [n = 244]	p
		Median (min-max) n (%)	Median (min-max) n (%)	
Antero-Posterior Diameter^a^, mm		7.6 (4.3-20.2)	**12.35 (3.8-58.5)**	**0.011**
Transverse Diameter^b^, mm		9.2 (6.4-32.0)	**17.1 (5.9-74.9)**	**0.010**
Longitudinal Diameter^c^, mm		10.1 (7.5-34.5)	**19.95 (8.4-74.1)**	**0.002**
TIRADS categories				0.339^d^
3		0 (0.0)	2 (0.8)	
4a		3 (18.7)	52 (21.3)	
4b		5 (31.3)	119 (48.8)	
4c		8 (50.0)	71 (29.1)	
Taller than wider shape		2 (13.3)	31 (12.9)	1.000
Absence of peripheral halo		13 (81.3)	176 (72.1)	0.568
Microcalcification		6 (37.5)	79 (32.4)	0.882
Macrocalcification		2 (12.5)	47 (19.3)	0.774
Echogenicity				
Isoechoic		2 (12.4)	**148 (61.2)**	**<0.001**
Hypoechoic		**5 (31.3)**	14 (5.7)	**0.003**
Iso-hypoechoic		9 (56.3)	80 (33.1)	0.106
Texture, Solid		15 (93.8)	236 (96.7)	0.441
Irregular margins		10 (62.5)	156 (63.9)	1.000
Vascularity, peripheral		2 (100.0)	18 (75.0)	1.000
Cytology				
	Non-diagnostic	0 (0.0)	**55 (22.5)**	**0.027**
	Benign	0 (0.0)	152 (62.3)	**<0.001**
	AUS/FLUS	4 (25.0)	25 (10.2)	0.089
	FN/SFN	2 (12.5)	6 (2.5)	0.081
	Suspicious for malignancy	**8 (50.0)**	5 (2.1)	**<0.001**
	Malignant	**2 (12.5)**	1 (0.4)	**0.010**
Lymphocytic thyroiditis in histopathology		**8 (50.0)**	45 (18.4)	**0.006**

TIRADS: Thyroid Imaging Reporting and Data System; AUS/FLUS: atypia of undetermined significance/follicular lesion for undetermined significance; FN/SFN: follicular neoplasm/suspicious for follicular neoplasm.

a,b,c n = 15 and n = 240 for malignant and benign, respectively.

d Monte Carlo simulation results based on 10000 samples.

The distribution of cytology and histopathology results in isthmic and non-isthmic nodules was shown in Suplementary Table. Among the nodules with non-diagnostic cytology and benign cytology, benign histopathology were significantly more frequent than those in the non-isthmic nodules (100% vs 93%, p=0.044; 100% vs 96.4%, p = 96.4; respectively). Within other cytology groups, there were no significant difference between isthmic and non-isthmic nodules with respect to the histopathology results (p > 0.05).

After non-diagnostic cytology results were excluded, when defining as benign cytology was “benign” or “negative”, and indeterminate (AUS/FLUS, FN/SFN, suspicious for malignancy) and malignant cytology results were “malignant” or “positive”, the sensitivity and specificity were obtained as 1.000 (95% CI: 0.806-1.000) and 0.803 (95% CI: 0.741-0.854) in isthmic nodules and 0.817 (95% CI: 0.780-0.850) and 0.832 (95% CI: 0.818-0.845) in non-isthmic nodules, respectively (Table 6). There were no significant differences between isthmic and non-isthmic nodules with respect to the sensitivity, specificity, and overall accuracy of the cytology (p > 0.05). However, the NPV of the cytology was significantly higher in isthmic nodules, the PPV was found as significantly lower in the isthmic nodules compared to those in non-isthmic ones (p < 0.05) (Table 6).

**Table 6:** Diagnostic accuracy measures of cytology in isthmic and non-isthmic nodules

Diagnostic accuracy measures	Isthmic nodules	Non-isthmic nodules	p
	(n = 204)	(n = 3255)	
Sensitivity (95% CI)	1.000 (0.806-1.000)	0.817 (0.780-0.850)	0.088
Specificity (95% CI)	0.803 (0.741-0.854)	0.832 (0.818-0.845)	0.310
Overall accuracy (95% CI)	0.819 (0.760-0.866)	0.830 (0.817-0.842)	0.681
PPV (95% CI)	0.302 (0.195-0.435)	0.451 (0.418-0.485)	**0.047**
NPV (95% CI)	1.000 (0.975-1.000)	0.964 (0.956-0.971)	**0.033**

PPV: positive predictive value; NPV: negative predictive value; CI: confidence interval.

## DISCUSSION

The clinical, ultrasonographical, and cyto-histopathological characteristics of isthmic and non-isthmic nodules were compared in the present study. Although isthmic nodules had lower malignancy rates compared to non-isthmic ones, US features (exception of macrocalcification), TIRADS categories and cytology results were similar between two groups. Furthermore, malignant isthmic nodules had similar US features, TIRADS categories and tumor characteristics as malignant non-isthmic nodules. Malignant isthmic nodules were smaller than benign ones. Isthmic nodules had higher NPV and lower PPV compared to non-isthmic nodules.

Prevalance of isthmic nodule was found as low compared to nodules located in lobes (7,11). US features associated with higher malignancy risk are primarily based on lober PTC (17) and there is limited data about US findings of isthmic nodules in the literature. In a study of small number of patients (16 benign and 12 malignant isthmic nodule), Goldfarb and cols. suggested that extend of operation could be more confidently predicted and planned when nodule evaluated with surgeon-performed US combined with FNAB findings in patients with isthmic nodule (14). Hahn and cols. studied with 48 patients with malignant isthmic nodule and 96 patients with malignant lober nodules. They found that isthmic nodules had more frequent circumscribed margin, a wider-than-tall shape, and ultrasonographical suspicion of extrathyroidal extension (tumor with capsular abutment of > 25% of its perimeter on ultrasound). First two are known as suggestive of benign features, but authors found them as predictors of malignancy in isthmic nodules. They suggested that these US characteristics appeared to have resulted from tumors growing in the thin isthmic space (17).

Nodule size was smaller in malignant isthmic nodules compared to benign isthmic nodules (14) although it was same in another study (27). The size of malignant isthmic nodules was found as significantly smaller than that of located left lobe in patients with metastasis (28). In a recent study with a large cohort, malignant nodules in isthmus were significantly smaller in size compared to that of middle, and lower, but not upper nodules of thyroid gland. They recommended that future consideration is given to adding a point to the guidelines for nodule location in the isthmus or using a lower size threshold for FNA or follow-up (7).

In the present study, 4.8% of nodules was located in isthmus and it was compatible with the literature. Almost all US features except for macrocalcification were similar in isthmic and non-isthmic nodules. It was noteworthy that when specifically isthmic nodules were evaluated, suspicious US features exception of hypoechogenicity, which was higher in malignant ones, were found as similar between malignant and benign ones, and malignant isthmic nodules had smaller size than benign isthmic nodules. The size data was compatible with the study of Goldfarb and cols. (14). These findings might support that the nodules in the isthmus, especially hipoechoic ones, should be evaluated with FNAB even if it is relatively small, as supported by the study of Jasim and cols. (7).

In our study, isthmic and non-isthmic nodules were found as similar regarding FNAB results. When malignant nodules were subdivided into two groups as isthmic and non-isthmic, any of the malignant isthmic nodules had non-diagnostic or benign results, but 14.3% and 15.8% of non-isthmic malignant nodules had non-diagnostic and benign results, respectively. Similarly, Goldfarb and cols. found that there was no nondiagnostic result in cytology of malignant isthmic nodule in their study in which isthmic nodules were evaluated in two groups as malignant and benign. Furthermore, they found that cytologies of all malignant isthmic nodules were malignant or suspicious for malignancy (14). Pastorello and cols. found that isthmic nodules had lower non-diagnostic results compared to lober nodules. They concluded that lower non-diagnostic results in isthmic nodules might be originated from easily accesible location of isthmus by biopsy due to the superficial location (10). Contrary to our result, Hahn and cols. did not find any significant difference between the two malignant groups (isthmic and non-isthmic) regarding the cytological findings. However, the rate of non-diagnostic result of isthmic nodule was higher in their study (2.1%) (17).

In the literature, malignancy rate in isthmus region was reported between 1% and 17.4% in different studies (7,9,10,12–21). In a study including 557 nodules with definitive cytologic diagnoses, although isthmic nodules had lower malignancy rate (2.5%), the rate was not significantly different in the isthmus, right (9.6%), or left lobe (7.2%) (8). Another study had shown higher malignancy rate in isthmic nodules (12.5%) compared to right (8%) and left (6.5%) lobes nodules but it did not also have statistical significance (9). On the other hand, in a largely cytology-based study, the malignancy rate of isthmic nodules was significantly higher than the right and left lobes (8.1%, 3.6%, 3.1%; respectively) (10). Similarly, in an another study, thyroid nodule location was found an independent risk factor in predicting thyroid cancer. Isthmic nodules had the highest risk of malignancy compared to right and left lober ones (17.4%, 10.1%, 9.6%; respectively). Of all malignant nodules, the malignancy rate was 10% for isthmic nodules (7). In the present study, malignancy rate of isthmic nodules was significantly low compared to non-isthmic nodules (6.2 % vs 12.5%, p = 0.002). Considering all malignant nodules, the proportion of isthmic nodules was 2.8%. The different malignancy rates in different studies could be due to selection bias of study population. In these studies, definition of isthmic nodule, FNAB criteria, extend of thyroidectomy, nodule diameter, and dominant nodule originating from isthmus or not were different. Some studies included only patients with dominant nodules and others had carcinomas outside the nodule (in the parenchyma).

Classical variant of PTC in isthmic nodules was significantly more frequent than those in lober PTCs (29). However, tumor size was smaller in isthmic nodules compared to non-isthmic nodules in some studies (12), it was reported as similar in other studies (21,30,31). We found similar PTC variant distribution and tumor size in two groups.

PTCs arising in the isthmus had increased rate of multifocality in some studies. It ranges from 27.2% to 67% (12,14,18,20). Authors attributed the multifocality both to the midline position of tumors, which may be caused more easily tumor spread to both lobes and may be due to fact of common intraglandular metastasis of PTC (20). We found similar multifocality rate between patients with malignant isthmic and non-isthmic nodules as in other studies (17,30).

Extrathyroidal extension is one of prognostic factors which is placed in TNM classification system (29). It is associated with local recurrence, lymph node metastasis, distant metastasis and disease-specific mortality (17). Isthmic PTCs were found to have more frequent extrathyroidal extension compared to lober ones in some studies (17,30,32). They concluded that it might be resulted from tumors growing in the thin istmus (12). Although it was similar in other studies (21,31), Wang and cols. (16) found lower extrathyroidal extension. Also they did not find a relationship between extrathyroidal extension and central lymph node metastasis. Capsular and adjacent tissue invasions were also found as increased in isthmic PTCs in comparison to PTC located in lobes (12,17,18,21). In the present study, extrathyroidal extension, capsular and lymphovascular invasions were similar in two groups.

Lymph node metastasis had shown a risk factor for recurrence and further metastasis (30). The most common site of lymph node metastasis in PTC is central compartment (33). The location of the tumor is associated with neck netastasis (34). The rate of cervical lymph node metastasis is increased in isthmic PTC with regard to PTC arising from thyroid lobes (18,21,27,28,30,32,35). It was also reported that clinical stage was similar in isthmic and non-isthmic PTCs (12,17,31). We found similar lymph node involvement and TNM staging between groups.

A possible linkage between thyroid cancer and lymphocytic thyroiditis has been suggested. Karatzas and cols. found similar rate of histopathological lymphocytic thyroiditis in malignant and benign isthmic nodules, but in malignant isthmic nodules it was lower than in malignant non-isthmic nodules (27). There were some studies which reported similar lymphocytic thyroiditis rates in malignant isthmic and malignant non-isthmic nodules (21,32). Although, we found that patients with isthmic nodules had lower lymphocytic thyroiditis rate compared to patients with non-isthmic ones, it was significantly higher in malignant isthmic nodules than benign ones.

According to our knowledge, there has been no study about comparing diagnostic accuracy measures in isthmic and non-isthmic nodules in the literature. We found that the sensitivity, spesificity and overall accuracy of the cytology were similar in two groups. While NPV was significantly higher, PPV was significantly lower in isthmic group in comparison with non-isthmic nodules. These results can be interpreted as isthmic nodules with indeterminant cytology are more likely to be benign compared to non-isthmic nodules. Although sample size was small, Goldfard and cols. reported that all of the patients with indeterminate cytology (n = 5) had benign histopathology (14). These results support that the follow-up or limited surgery might be considered for the management of indeterminate isthmic nodules.

The strength of our study was that, to our knowledge, on this topic, it had the most nodules whose diagnoses were confirmed histopathologically in the literature. Additionally, our study extensively examined demographic, ultrasonographic and cyto-histopathological features. However, retrospective design which could be a possible reason of selection bias for study population was a limitation of our study.

In conclusion, isthmic nodules have almost similar US and cytopathological features, and tumor characteristics to non-isthmic ones. They seem to be indolent because they had lower malignancy rate compared to non-isthmic nodules. Patients with isthmic nodule and indeterminate or nondiagnostic cytology might be candidates for follow-up. Careful examination of relatively small and hypoechoic nodules in the isthmus might be required due to the possibility of malignancy.

## Figures and Tables

**Table:** The distribution of cytology and histopathology results in isthmic and non-isthmic nodules

Cytology	Isthmic nodules						Non-isthmic nodules						p^2^
	Malignant histopathology			Benign histopathology			Malignant histopathology			Benign histopathology			
	n	%*	%ǂ	n	%*	%ǂ	n	%*	%ǂ	n	%*	%ǂ	
Nondiagnostic	0	0.0	0.0	55	**22.5**	100.0	78	14.2	6.6	1095	**28.2**	93.4	**0.044**
Benign	0	0.0	0.0	152	**62.3**	100.0	86	15.7	3.6	2316	**59.7**	96.4	**0.032**
AUS/FLUS	4	25.0	13.8	25	10.2	86.2	99	**18.0**	21.6	360	9.3	78.4	0.447
FN/SFN	2	12.5	25.0	6	2.5	75.0	27	**4.9**	32.5	56	1.4	67.5	>0.999
Suspicious for malignancy	8	**50.0**	61.5	5	2.1	38.5	126	**23.0**	73.3	46	1.2	26.7	0.351
Malign	2	**12.5**	66.7	1	0.4	33.3	133	**24.2**	95.7	6	0.2	4.3	0.142
p^1^	**<0.001**						**<0.001**						

AUS/FLUS: atypia of undetermined significance/follicular lesion of undetermined significance; FN/SFN: follicular neoplasm/suspicious for follicular neoplasm.

* column proportion;

ǂ row proportion.

1 test result for comparing the distribution of cytology between malign and benign nodules within isthmic and non-isthmic nodules.

2 test result for comparing the distribution of histopathology between isthmic and non-isthmic nodules within each cytology group.

Bold column proportions are significantly higher in the corresponding histopathology.
